# Proteomic analysis of honeybee worker (*Apis mellifera*) hypopharyngeal gland development

**DOI:** 10.1186/1471-2164-10-645

**Published:** 2009-12-31

**Authors:** Mao Feng, Yu Fang, Jianke Li

**Affiliations:** 1 Institute of Apicultural Research, Chinese Academy of Agricultural Science/Key Laboratory of Pollinating Insect Biology, Beijing 100093, PR China

## Abstract

**Background:**

Hypopharyngeal glands (HG) of honeybee workers play an important role in honeybee nutrition and caste differentiation. Previous research mainly focused on age-dependent morphological, physiological, biochemical and genomic characters of the HG. Here proteomics and biochemical network analysis were used to follow protein changes during the HG development.

**Results:**

A total of 87, 76, 85, 74, 71, and 55 proteins were unambiguously identified on day 1, 3, 6, 12, 15 and 20, respectively. These proteins were major royal jelly proteins (MRJPs), metabolism of carbohydrates, lipids and proteins, cytoskeleton, development regulation, antioxidant, molecule transporter, regulation of transcription/translation, proteins with folding functions. The most interesting is that MRJP's that have been detected in the HG of the newly emerged worker bees. The MRJP's expression is at peak level from 6-12 days, was validated by western blot analysis of MRJP1, 2 and 3. Moreover, 35 key node proteins were found in the biochemical networks of the HG.

**Conclusions:**

HG secretes RJ at peak level within 6-12 days, but the worker bee can secrete royal jelly (RJ) since birth, which is a new finding. Several key node proteins play an important role in the biochemical networks of the developing HG. This provides us some target proteins when genetically manipulating honeybees.

## Background

The hypopharyngeal gland (HG) of the worker bee is a paired long tuberous organ connected to many acini each of which is composed of about a dozen secretory cells [1]. The HG secretes a proteinaceous substance which is fed to larvae, queens and drones. It has attracted much attention in research, not only for its most striking feature which is it's age dependent role associated with the shifting of tasks from nursing to foraging worker bees [2], but also for its importance in royal jelly (RJ) production [3,4]. Morphologically, acini size of the HG radically changes with age [5]. Peak size is found at around 6 days in bees during the summertime, when workers are known to feed the larvae with RJ [6], but it begins to decrease after day 15. The volume of the acini and the number of secretory vesicles decreases, and no vesicles are visible after 3 weeks of age. It has also been reported that the HG size is positively correlated with gland activity [5].

Physiologically, the normal course of the development of these glands (sizes of acini) is well described [7]. The glands are well developed during the stage of ontogenesis when the individuals are acting as nurse bees and they degenerate when these individuals become foragers, although at the beginning of their foraging career bees can still have well developed glands [8]. The HG is fully developed and shows high rates of protein synthesis in nursing bees, but regresses in foraging bees [9]. It is believed that the HG exists in two distinctly differential states, first producing RJ for brood nutrition, followed by enzyme production, e.g. age-polymorphism [10].

Biochemically, in accordance with the age-dependent role, the glands express a specific gene for the 64 kDa protein (major royal jelly protein 3, MRJP3) in the nurse bee, while the gene for α-glucosidase is expressed in the forager bee glands only [11]. Some reports also confirm that foragers could produce α-glucosidase [12,13], which increases with the age of workers [5], glucose oxidase [2], and other enzymes such as galactosidase, esterase, lipase and leucine arilamidase [5] to process nectar into honey. The expression of these carbohydrate-metabolizing enzymes in the HG is age-dependent in worker honeybees [10]. In contrast, the gene coding for a 56 kDa protein is expressed in both the nurse bee and forager bee glands [10]. Santos et al [13] identified the protein complement of HG of Africanized nurse bees (*Apis mellifera *L.) and almost all were related to the MRJP family and associated with the metabolism of carbohydrates and energy.

Several morphological, physiological and biochemical approaches have been documented in the study of the honeybee HG development. However, these findings are mainly derived from conventional and/or transcriptional data reflected by expressed genes. Since changes in the protein level in many organisms are not accompanied by altered level of transcription, previous research on the HG development could not describe the entire picture well of this fascinating developmental problem. Proteomics, a global strategy for mining the protein information, is of great importance for developmental studies. The recent sequenced honeybee (*Apis mellifera *L.) genome and transcriptome [14] has stimulated new efforts in investigating the proteome profile during the gland development. The aim of the present study was to obtain a protein profiling of the HG development on a global scale, thus making possible a leap from the study of individual genes or proteins to an integrated understanding of how gene networks enable complex functions to be carried out. This leap has the potential to exponentially expand our knowledge of honeybee biology.

## Results

### Protein profiling of HG at different developmental phases

**Figure 1:**
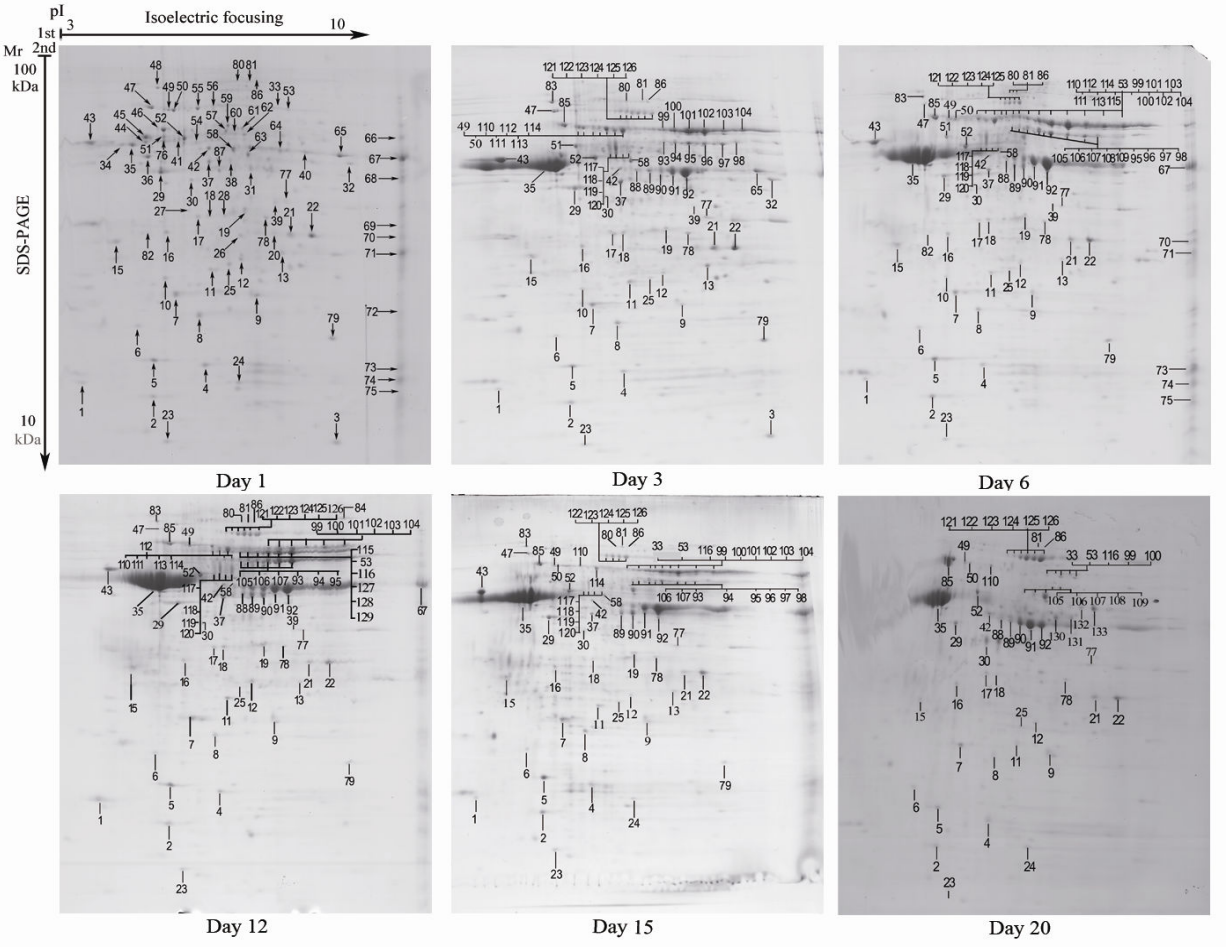
**2-DE profile of hypopharyngeal gland of honeybee workers (*Apis mellifera *L.) on day 1, 3, 6, 12, 15 and 20, respectively**. 2-DE profile of hypopharyngeal gland of honeybee workers (*Apis mellifera *L.) on day 1, 3, 6, 12, 15 and 20, respectively. 200 *μ*g of each sample were subjected to 2-DE and stained by CBB G-250. Number-labeled spots were cut out and subjected to tryptic digestion for mass spectrometry analysis.

Protein samples from 6 different points of time, occurring during the development of HG of honeybee workers were analyzed by 2-DE. Figure 1 is a representative gel image of the best 5 runs, showing the soluble proteins extracted from the HG of honeybee workers on day 1, 3, 6, 12, 15 and 20. Using the PDQuest analysis software, a total of 175, 189, 209, 190, 168 and 151 proteins were detected at 6 time points, respectively, with *M*r encompassing from 14.40 to 102.56 kDa and p*I *from 4.30 to 9.78. Among them, 51 proteins were resolved in all images at each developmental phase with *M*r and p*I *in the range of 17.3~93.4 kDa and 4.60~9.15, respectively. Besides, there were 28, 15, 10, 13, 22 and 18 proteins uniquely expressed on day 1, 3, 6, 12, 15 and 20, respectively (Figure 1). Totally, 87, 76, 85, 74, 71 and 55 proteins were identified successfully at each time point, respectively, their detailed information is shown in supplementary Table S in Additional File 1. The protein spots remained unidentified were mainly attributed to their values being too low to produce a spectrum, or because the C. I. % of the database search was not higher than 95% in order to yield unambiguous results.

**Figure 2:**
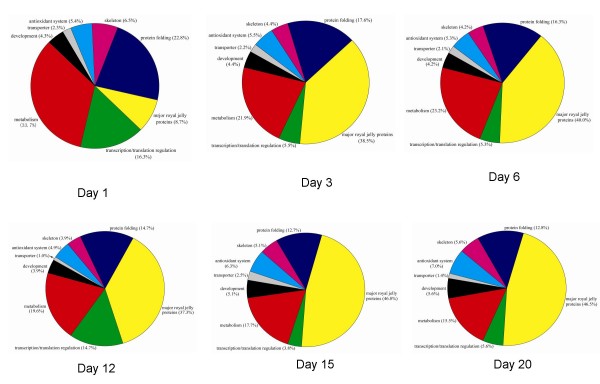
**Functional classification of the identified proteins from day 1 to day 20 of the hypopharyngeal gland of the honeybee workers (*Apis mellifera *L.), respectively**.

Among the identified proteins, the most present form was found to be MRJPs increasing from 8.7~46.8% from day 1-day 20. The second largest category was related to the metabolism of proteins, lipids, nucleic acids and carbohydrates decreasing from 33.3~15.5%, of which proteins related to metabolism of carbohydrates were the most present form. The third largest group was the proteins with folding functions, decreasing from 22.8~12.7%, of these heat shock proteins (Hsps) were the highest in number. While the proteins that regulation of transcription and translation process were presented most on day 1 and day 12 accounting for 14.0~16.0%, on the other days it was presented at a rate of 3.8~5.6%. And developmental regulating factors were represented from 3.9~5.6%. Additionally, skeleton, antioxidant and transporter proteins were 3.9~6.5%, 4.9~7.0% and 1.0~2.3%, respectively (Figure 2).

### Expressional analysis of the identified proteins

**Figure 3:**
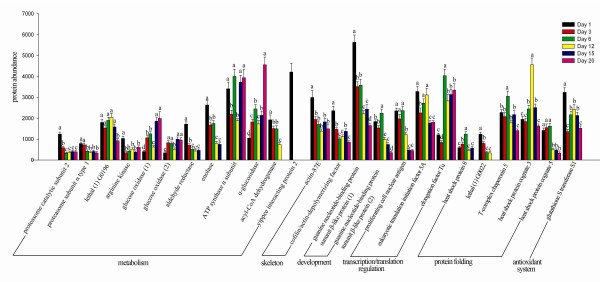
**Proteins differentially expressed on day1 to day 20 of the hypopharyngeal gland of the honeybee workers (*Apis mellifera *L.), respectively**. Different lower case letters (a, b, c, d) above the bars indicates significant differences between day 1 to day 20 of the hypopharyngeal gland (*p *< 0.05), correspondingly. A is significantly higher than b, c and d, b is significantly higher than c and d, c is significantly higher than d.

Based on the statistical analysis of the identified proteins, 12 proteins differentially expressed in the metabolism of proteins, lipids, nucleic acids, carbohydrates, 7 proteins, i. g. proteasome subunit α type 1 (spot 18), proteasome catalytic subunit 2 (spot 25), lethal (1) G0196 (spot 2), arginine kinase (spot 30), acyl-CoA dehydrogenase (spot 39), enolase (spot 58), aldehyde reductase (spot 19), yippee interacting protein 2 (spot 68) expressed higher at early stages of the HG development, while glucose oxidase (two isoforms, spots 81 and 86) and α-glucosidase (spot 85) related to digesting nectar into honey, expressed higher at later stage of the HG development. In addition, the proteins that form the skeleton (Actin 87E, spot 29; Cofilin/actin-depolymerizing factor, spot 4), regulation of development (guanine nucleotide-binding protein subunit β-like protein, two isoforms, spots 21 and 22), transcription and translation (proliferating cell nuclear antigen, spot 15; eukaryotic translation initiation factor 5A, spot 5; elongation factor Tu, spot 77) decreased along with the HG development. And the proteins with folding functions (heat shock protein 8, spot 49; T-complex chaperonin 5, spot 54; heat shock protein cognate 5, spot 55) had a higher expression on day 6 (Figure 3). MRJP1 (spot 35) expressed significant higher on day 6 and day 12 than those other days. Among the altered expression of MRJP2, 5 isoforms (spots 88-92) expressed at higher level on day 6, and 3 major isoforms of MRJP3 (spots 100, 102 and 120) expressed high level on day 6 and day 12.

**Figure 4:**
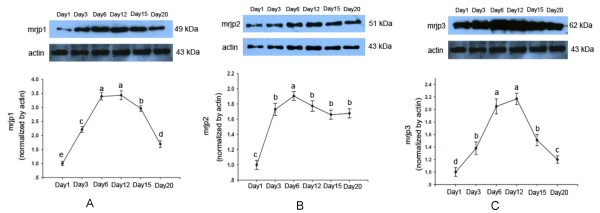
**Western blot analysis of major royal jelly protein1 (MRJP1) (A), MRJP2 (B) and MRJP3 (C)**. Whole cell lysates from hypopharyngeal gland on day 1, 3, 6, 12, 15 and 20 of honeybee worker (*Apis mellifera *L.) were subjected to SDS-PAGE followed by western blot analysis. MRJP1, 2 and 3 were detected using polyclonal antibody anti-MRJP1, 2 and 3, respectively. Equal loading of lanes was maintained by performing a total protein assay and is confirmed by western blot analysis using an anti-actin antibody. Upper panel, western blot analysis; lower panel, relative densitometry analysis (normalized by actin). A is significantly higher than b, c and d, b is significantly higher than c and d, c is significantly higher than d.

To further validate the change of protein abundance at different developmental stages, MRJP1, MRJP2 and MRJP3 were selected and applied to western blot analysis as shown in Figure 4. The band volume was measured by Quantity One (Bio-Rad Laboratories Ltd.). It was clear that MRJP1, MRJP2 and MRJP3 significantly increased their expression just from immediate emergence to 6-day-old workers, and then decreased after day 12. But the expression of these proteins was at peak level during day 6-day 12 (Figure 4). This was consistent with the results of 2-DE generally.

### Network analysis

**Figure 5:**
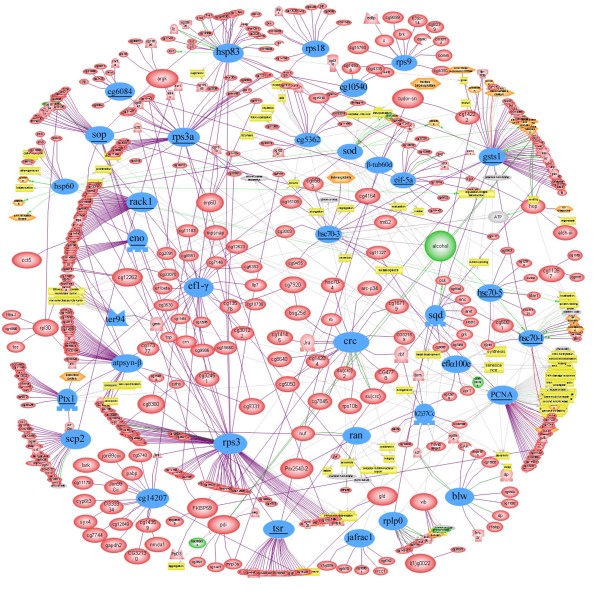
**Network analysis of all the pathways and interactions connected to all the identified proteins (n = 133) and 27 differentially expressed proteins**. Those highlighted in blue represent the key node proteins (n = 35) identified in this study and revealed by the network software program. Meanwhile, underlined proteins represent the deferentially regulated proteins. Protein entities which belong to different functional groups were represented as different shapes according to the default settings of the software, "sickle" "red elipse" for proteins, "sickle" for kinases, "rhomb" for ligands, "stick" for receptors, "O-vertex" for transcription factors, "2 triangles" for phosphates, "sticks" for receptors, "grey elipse" for cell objects, "orange hexapon" and "yellow rectangle" for cell process.

Since proteins perform their functions together in networks, we analyzed all the pathways and interactions connected to all the identified proteins hoping to find the possible key node proteins during the HG development using pathway studio software. It showed that 35 of the identified proteins acted as key node proteins. These proteins were 7 proteins related in metabolism of carbohydrate and energy production, i. g. aldehyde reductase (CG6084), transitional endoplasmic reticulum ATPase (ter94), sterol carrier protein X-related thiolase (scp2), ATP synthase β subunit (atpsyn-β), ATP synthase α subunit (blw), enolase (eno) and malate dehydrogenase (CG5362); 7 proteins with folding functions, i. g. calreticulin (crc), heat shock protein (hsp) 90-α isoform 2 (hsp83), hsp 8 (hsc70-1), 60 kDa hsp (hsp60), small Hsp20-like chaperone (CG14207), hsp cognate 5 (hsc70-5) and hsp cognate 3 (hsc70-3); 3 proteins related to antioxidant activities, superoxide dismutase (sod), glutathione S transferase S1 (gsts1) and thioredoxin peroxidase 1 (jafrac1); 2 proteins involving in development, guanine nucleotide-binding protein subunit β-like protein (rack1) and lethal (2) 37 Cc (l(2)37 Cc); 4 cellular skeleton proteins, cofilin/actin-depolymerizing factor (tsr), act 87E (ptx1), β-tubulin (β-tub60d) and F-actin capping protein α subunit (CG10540); 12 proteins acting as transcription and translation regulators, shown as ribosomal protein S18 (rps18), 40S ribosomal protein S3a (rps3a), 40S ribosomal protein S9 (rps9), 40S ribosomal protein S2 (sop), 60S acidic ribosomal protein P0 (rplp0), RNA-binding protein 3 (sqd), 40S ribosomal protein S3 (rps3), eukaryotic translation initiation factor 5A (eif-5a), elongation factor 1-alpha (ef1-α), eukaryotic translation elongation factor 1-γ (ef1-γ), proliferating cell nuclear antigen (PCNA) and GTP-binding nuclear protein Ran (ran) (Figure 5).

## Discussion

This study employed a systematic analysis of the whole time-range of the honeybee HG at the protein level, in parallel with metabolic pathway approaches. Some of the biochemical pathways were built up where 35 key node proteins were found in all the identified proteins. To our present knowledge, this is the first large scale and all around analysis of the development of HG in this model organism. The results provide a global perspective on the protein and the biochemical pathways involved in the HG development of the honeybee workers, helping us to better understand honeybee biology. Obviously, some different spots in gels were identified to be the same proteins in supplementary Table S (Additional File 1). They were isoforms and their different distributions in gels might be caused by some post-translational modifications (PTMs), such as phosphorylation and possibly alternative splicing that resulting in a shift of *M*r and p*I *of these proteins.

The HG of honeybee workers goes through the undeveloped, developed and regressive phases accompanying the shifting of tasks from nursing to foraging within 20 days in a normal colony. In young workers, usually not more than 13 days past eclosion, the HG, which is fully developed and shows high rates of protein synthesis in nursing period, is already secreting RJ for brood breeding. Eighteen days later, when workers become foragers, the HG begins to regress and produce enzyme products to digest nectar into honey [2,10]. In the present study, 6 time points were chosen in an attempt to distinguish proteins expressed at different time points in the whole range of the HG development. Based on the proteomic profile, we could clearly observe that 6 and 12 day-old HGs expressed more proteins than that of the others. The proteins that were identified in each time point varied greatly, and belong to a broad range of different classes and functional pathways (Figure 2), implying a wide range of proteins are necessary for the HG development.

RJ, secreted from the HG and mandibular gland of the worker honeybee [15], is the exclusive food for the queen honeybee and young larva [16]. Theoretically, MRJPs, including MRJP1, 2, 3, 4 and 5, should be the main contents in the HG. The identified MRJPs in this study have validated these assumptions. Attributed to the potential glycosylation sites and extensive repetitive regions in the C-terminal of the proteins [17], some isoforms of MRJP2 and MRJP3 were present in this study. Interestingly, MRJPs were identified in the 1-day-old HG and 3-day-old HG had a typical pattern of RJ (Figure 1). This suggests that the newly emerged honeybee workers are likely have the ability to secrete RJ and start to produce RJ much earlier than expected. It was confirmed by western blot that MRJP1, 2 and 3 could be detected from day 1, and by day 3 express at significantly higher levels than those of day 1 (Figure 4A-4C). Furthermore, the HG secreted RJ at peak level from 6 to 12 days, which could also be confirmed by western blot analysis (Figure 4). These findings show that the period from 6 to 12 days is the peak time for the workers to secret RJ and nurse the brood. After that the output of RJ begins to decrease because of the shifting tasks and the shrinkage of the HG [9].

Eighteen proteins were related to the metabolism of carbohydrate and energy production involved in glycolysis (spots 13, 32, 58, 63 and 87), citric acid circle (spot 78), and ATP generations (spots 1, 3, 19, 30, 31, 36, 40, 48, 57, 60, 65 and 66) (supplementary Table S in Additional File 1) in the present study. Seven of them were detected as key node proteins (CG6084, atpsyn-β, blw, ter94, eno, scp2 and CG5362) (Figure 5). Both the higher number and most of the differentially expressed were in the early stages, suggesting that not only the development of the HG needs much metabolic energy for cell divisions and secretory synthesis in this early period, but also the honeybee has an evolutionary strategy to cope with the selective pressure on its extremely carbohydrate-rich diet [18,19]. The down-regulated of these proteins in the latter period are coincided well with *Drosophila *that gene encoding enzymes involved in energy metabolism are down-regulated during the late larval ecdysone pulse [20]. Acyl-CoA dehydrogenase (spot 31) is involved in mitochondrial fatty acid β-oxidation, which fuels hepatic ketogenesis during prolonged fasting and periods of higher energy demands [21]. The down-regulated here is probably due to the honeybee worker dropping its lipid storage dramatically prior to the onset of foraging. The main task for the forager bee is to collect nectar and divert it into honey by adding some enzymes, such as α**-**amylase (spot 79) and α-glucosidase (spot 85). These transferases could digest polysaccharides, disaccharides into monosaccharide. The up-regulated of α-glucosidase (Figure 3) coincides well with the task changes of the honeybee workers. The expression of glucose oxidase (GOD) in this study (spots 80, 81 and 86) is likely used for the biological production of gluconic acid and for the removal of either glucose or oxygen from foodstuffs in order to improve their storage capability [22]. So the up-regulated of this acid is also related to the work of the workers.

Hsps function primarily as a molecular chaperone, facilitating the protein folding, preventing protein aggregation, or targeting improperly folded proteins in specific degradative pathways [23]. The third largest group in this study was related to proteins with folding functions, of which Hsps were the most represented forms. Six of them, hsp83 (spot 83), hsc70-1 (spot49 and 50), hsp60 (spot 46 and 51), CG14207 (spot 10), hsc70-5 (spot 55) and hsc70-3 (spot 47), were the key node proteins for the HG development (Figure 5). In honeybees, the enhanced expression of Hsps and cell death in the midgut of the bee's larvae infected with *Paenibacillus larvae *or *Bacillus larvae *[24,25], the larval salivary glands treated with acaricides [26] and under heat stress conditions [27] have been well documented, which could be a defense mechanism to prevent against stress tolerance. Hsps have widely been identified in honeybees, including worker larvae [3], the embryos [19], the head and brain of the workers [28], the hemolymph [29] and the venom gland [30], respectively. The higher number of Hsps identified in the early stage (Figure 2) and higher abundance expressed from day 6 to day 12 (Figure 3) suggests that they probably act as molecular chaperones by assisting in the correct folding of nascent proteins, thus contributing to cell maintenance and protein (RJ) secretory activity of the HG since our experiment was carried out under normal physiological conditions.

The present identified antioxidant proteins involving jafrac1 (spot 8), thioredoxin reductase-1(spot 64), sod (spot 24), gsts1 (spot 7) and peroxiredoxin 2540 (spot 11), could also been found in the honeybee embryo [19], the venom gland [30] and the HG of wintering honeybee workers [31]. Among them, gsts1, jafracl and sod played the key role for the antioxidant system in the developing HG (Figure 5). Sod, thioredoxin reductase and peroxiredoxin 2540 are able to metabolize peroxides [32,33]; and Jafracl, a superfamily of detoxication protein, can change xenobiotic to harmless products [34]. The oxidative damage caused by the reactive oxygen species (ROS) is to be intensified with the increase of ROS due to a high demand for oxygen in fast growing organism [35]. Therefore, the HG during the nursing period is fully developed and shows high secreting activity, which means the metabolism in this stage is vigorous; on the contrary, the metabolism is slowed down in the HG of foraging bees because of the regression and low activity of the HG [10]. As a result, the up-regulated of gstsl in the early stage, followed by a down-regulated in the late stage, is responsible for the changes with the development of the HG in this study.

The cytoskeleton plays important roles in both intracellular transport and cellular division. Among the identified cytoskeletal proteins, β-tub60d (spot 34), F-actin capping protein α subunit (spot 16), tsr (spot 4 and 6), ptx1 (spot 29) were the key node proteins in the cytoskeletal system (Figure 5). Among those key node proteins, tsr and ptx1 showed a significantly down-regulated trend (Figure 3)**. **Actin plays an important role during dorsal closure throughout the embryonic development in *Drosophila *[36], and tsr, which restricts the actin polymerization [37], appears to have control of actin-based motility processes and enhances removal of ADP bound actin monomers from the pointed end of an actin filament [38]. Down-regulated of them in the latter developmental phase coincides with the secretory activity and the acini size of the HG beginning to decrease after 15 days when workers becoming foraging bees [5,9], cell death predominates in the latter foraging period [39].

Obviously, the development of HG requires the presence of growth factors to ensure its development. Rack1 (spots 21 and 22) and l(2)37 Cc (spot 12), acting as key node proteins, were the growth factors in the HG development of the honeybee workers. In *Drosophila*, rack1 is expressed at all developmental stages and in many tissues. It is essential at multiple steps of *Drosophila *development, particularly in oogenesis [40]. Lethal (2) 37 Cc is required for larval metabolism from larvae to pupae, and is expressed in early embryos, late embryos, late third instar larvae and adults of the *Drosophila *[41]. Overall, the down-regulated of rack1 (Figure 3) suggests that a decrease in the secretory activity and lower growth factor titers are demanded with the regression of the HG.

A group of proteins were identified as being responsible for the initiation of translation and accuracy of elongation, with 12 were the key node proteins (Figure 5). PCNA (spot 15), a highly conserved protein, is an essential component in the DNA replication and DNA repair [42]. It is also required for some repair pathways, including NER [43] and mismatch repair [44]. In *Drosophila*, temperature shift studies reveal that the vital function of PCNA is required throughout virtually all stages of fly development [44]. Eif-5a (spot 5) is involved in the first step of peptide bond formation in translation and is essential for cell proliferation and cell-cycle regulation [45]. While ef-1α (spot 67) is a nuclear protein coding gene involved in the GTP-dependent binding of charged tRNAs to the acceptor site of the ribosome during translation. In *Drosophila*, ef-1α is expressed at different times during development [46]. At the same time, several ribosome proteins (Rps) (spots 28, 69, 70, 71, 72, 73 and 75) were identified involving in the cellular process of translation [47]. In addition to their ubiquitous expression, these proteins are present simultaneously and in essentially fixed ratios in the ribosome and in the cell as a whole. It has shown that disrupting ribosome function can result in an array of fascinating dominant phenotypes in *Drosophila *[48]. Rps3 (spot 71) is very crucial for translation as a component of the 40S ribosomal subunit, and also acts as a damage DNA endonuclease [49]. Sop (spot 70) mainly participates in aminoacyl-transfer RNA binding to the ribosome, potentially affecting the fidelity of mRNA translation [50]. Rplp0 (spot 28) plays an important role in polypeptide chain elongation during translation. Most of the Rps identified in this study that have been documented in ribosomal proteome of the *Drosophila *[51]. Newly synthesized ribosomal proteins are found in association with ribosomal subunits as soon as 90 min after fertilization of *Drosophila *embryos [52]. The ribosome number is thought to control cellular growth [53], in this regard, the fast growing cells need more Rps to insure their growth. The expression of Rps in this study did not increase within 6 to 12 days might attribute to the increased cell proliferation does not always correlate with the higher expression level of ribosomal [54]. Some ribosomal proteins may have more specific roles in regulating proliferation than simply influencing the rate of protein biogenesis. As such, their increased expression may be a separate phenomenon from the general increase in the synthesis of ribosomal proteins in dividing cells [55]. In general, the highest key node proteins in this group that altered their expression are likely to work together to regulate the process of transcription and translation for the regular development of the HG and RJ secretion.

## Conclusions

In this study, we used proteomic and biochemical network analysis to describe the protein profile of HG within the whole range of the development. A number of proteins were successfully identified and several key node proteins were analyzed. These proteins were involved in multiple functions and took part in a variety of biological processes. The results not only expand our knowledge of honeybee biology, but also provide us some target proteins when genetically manipulating this model insect. The present study found molecular evidence that the newly emerged worker bees could secrete royal jelly. Behavior observation should be conducted to test that whether these workers have the ability to nurse brood when they emerge from the comb cells. It would be better if the proteomics data could be verified by the transcriptome profile of the HG. This would give a more comprehensive understanding of the HG development.

## Methods

### Honeybee

Mated Italian honeybee queens (*Apis mellifera *L.) were bought from Pisa University, Italy and introduced into honeybee colonies for egg laying at the Institute of Apicultural Research, Chinese Academy of Agricultural Science. Worker bees were marked with paint on their thorax when just emerging from the cells and then placed back into colonies. Sixty of the marked workers from 5 colonies were collected on day 1, day 3, day 6, day 12, day 15 and day 20 in June, 2008, respectively, then anesthetized on ice and the HG was dissected using a binocular microscope.

### Chemical regents

Urea, Tris-base, sodium dodecyl sulfate (SDS), sodium bicarbonate ((NH4)HCO_3_), dithiothreitol (DTT), iodoacetamide and bovine serum albumin (BSA) were purchased from Sigma (St. Louis, MO, USA). Bio-lyte was from Bio-Rad (Hercules, CA, USA). Acrylamide, N,N'-methylenebisacrylamide, ammonium persulfate (AP), N,N,N',N'-tetramethylethylene diamine (TEMED), 3-[(3-cholamidopropyl)-dimethylammonio]-1-propane sulfonate (CHAPS), glycerol, Bromophenol Blue, Coomassie Brilliant Blue (CBB) G-250 and Tween-20 were obtained from Amresco (Solon, Ohio, USA). α-cyano-4-hydroxycinnamic acid (CHCA) was obtained from Bruker Daltonics (Billerica, Mass. USA). Trypsin was purchased from Roche (Modified, Sequencing Grade, Roche, Mannheim, Germany). Trifluoroacetic acid (TFA) and acetonitrile were from J. T. Baker (Phillipsburg, NJ, USA).

### Protein extraction and two-dimensional gel electrophoresis (2-DE)

Protein extraction was performed as our previously described method [4]. Protein concentration was determined according to the method developed by Bradford using BSA as the standard. The absorption was measured at 595 nm (Beckman, spectrophotometer DU800). A 200 μg protein sample was suspended in lysis buffer (LB) and then mixed with a rehydration buffer (containing 8 M urea, 2% CHAPS, 0.001% Bromophenol Blue, 45 mM DTT, 0.2% Bio-lyte pH 3-10). The mixture was loaded on a 17 cm IPG strip (pH 3-10, linear, Bio-Rad Hercules, CA, USA). IEF was performed at 18°C (PROTEAN IEF Cell,Bio-Rad Hercules, CA,USA) according to the following program: active rehydration for 14 h at 50 V; 250 V for 30 min × 4 times; 1000 V for 60 min; 9000 V for 5 h;9000 V for 60,000 Vh. Before SDS-PAGE, the IPG strips were first equilibrated for 15 min in equilibration buffer 1 (6 M urea, 0.375 M Tris-HCl [pH 8.8], 20% glycerol, 2% SDS, 2% DTT) and then continued in equilibration buffer 2 (6 M urea, 0.375 M Tris-HCl [pH 8.8], 20% glycerol, 2% SDS, 2.5% iodoacetoamide) for 15 min. After the equilibration, the strip was transferred to SDS-PAGE gel, 12% T separating gel (1.00 mm). Meanwhile, 10 μL of 2-DE marker was loaded into a piece of filter paper, and then it was transferred adjacently to the acid tip of the strip when the filter paper was nearly dry. The second dimension electrophoresis, SDS-PAGE, was performed on PROTEAN xi Cell (Bio-Rad Hercules, CA, USA) at 25 mA/gel for about 6.5 h. The gels were stained with CBB G250 and scanned with transparent model, then analyzed with PDQuest V 8.0 (Bio-Rad Hercules, CA, USA). Each sample was replicated four times and the best five with good reproducibility were subjected to analysis.

### Image acquisition and statistics analysis

Gels were fixed overnight in 50% (v/v) ethanol with 10% (v/v) acetic acid, washed with water, and stained with CCB G-250. The best five reproducible gels from the samples with 3 replications were subjected to analysis. Data from 2-DE gels were analyzed using PDQuest V 8.0 (sensitivity 6.86, scale 9). The authenticity and outline of each spot were validated by visual inspection and edited when necessary.

Spot quantities between gels often have some variation in spot size and intensity, due not to differential protein expression, but rather to other variations within each gel. In order to accurately compare the gels, the intensity of each protein spot was normalized relative to the total abundance of all valid spots. After normalization and background subtraction, a matched set was created for all samples. The program generated a quantitative table with all normalized optical spot densities that permitted an analysis of variance (ANOVA) to detect statistical differences between the measurements of the same spot in all replicate samples. An ANOVA (Version 6.12, SAS Institute, Cary, N.C., USA), a Duncan parametric test, was used to test the significance of the normalized volume in total density of identified proteins in all gels. An error probability of *p *< 0.05 was considered to be statistically significant.

### Tryptic digestion for mass spectrometry

The CBB stained spots were excised and destained for 30 min using 100 mL acetonitrile (50%) and 25 mM (NH4)HCO_3 _pH 8 (50%) for 3-4 times until the gel was transparent with no color, dried for 10 min with acetonitrile (100%). The gels were dried for 30 min using a Speed-Vac system. Then 2.5 mL of 25 mM (NH4)HCO_3 _was added to the 25 μg trypsin (final concentration 10 ng/μL); 10 μL of this solution was pipetted on each dried protein spot and incubated for 60 min at 4°C. The supernatant was discarded to minimize auto digestion of trypsin. Then the sample was incubated for 14 h at 37°C. To extract the peptide fragments from the tryptic digests, 20 μL of 5% (v/v) TFA were added and incubated for 60 min at 37°C. Thereafter, 20 μL of 50% (v/v) acetonitrile [containing 2.5% (v/v) TFA] acid were added to the gel and incubated for 60 min at 30°C. After each step the supernatants were pooled and dried using a Speed-Vac system.

### MALDI-TOF/MS and MALDI-TOF-TOF/MS analysis

Before obtaining the mass spectra of the peptide mixture, the digested peptides were desalted and cleaned with ZipTip C18 pipette tips (Millipore Corp., Bedford, MA, USA) according to the manufacturer's instructions. All analyses were performed using a Bruker Daltonics Autoflex (Bruker Daltonics Billerica, Mass. USA) operated in the delayed extraction of 190 ns and reflector mode with an accelerating voltage of 20 KV. The peptide mixture was analyzed using a saturated solution of CHCA in 50% acetonitrile/0.1% TFA. External calibration was performed with a peptide calibration standard (Bruker Daltonics Billerica, Mass. USA, Part No.: 206195) and internal calibration with trypsin autoproteolytic fragments. Finally, the masses of proteolytic peptide fragments, were obtained by peptide mass fingerprinting (PMF), a mass spectrometry based protein identification technique.

Some proteins were analyzed by MALDI-TOF-TOF/MS. The tryptic mixed peptides from 2D-PAGE gels were loaded onto an AnchorChip (Bruker Daltonik Billerica, Mass. USA) target plate followed by adding matrix solution CHCA (4 mg/mL) in 70% acetonitrile/0.1% TCA. The loaded target was analyzed in an Ultroflex MALDI-TOF-TOF MS (Bruker Daltonik Billerica, Mass. USA) equipped with a nitrogen laser (337 nm) and operated in reflector/delayed extraction mode for MALDI-TOF PMF or LIFT mode for MALDI-TOF-TOF with fully automated mode using the FlexControl software (Bruker Daltonik Billerica, Mass. USA). An accelerating voltage of 25 kV was used for PMF. Calibration of the instrument was performed externally with (M+H)+ ions of angiotensin II, angiotensin I, substance P, bombesin, ACTH clip 1-17, ACTH clip 18-39, and somatostatin 28. Each spectrum was produced by accumulating data from 100 consecutive laser shots, and spectra were interpreted with the aid of the MASCOT software (Matrix Science, London, UK). The peaks with S/N >5, resolution >2500 were selected and used for LIFT-TOF/TOF-MS/MS from the same target. A maximum of six precursor ions per sample were chosen for MS/MS analysis. In TOF1, all ions were accelerated by 8 kV under conditions promoting metastable fragmentation. After selection of jointly migrating parent and fragment ions in a timed ion gate, the ions were lifted by 19 kV to a high potential energy in the LIFT cell. After further acceleration of the fragment ions in the second ion source, their masses could be analyzed simultaneously in the reflector with high sensitivity. The spectra were processed using FlexControl and BioTools 2.2 software tools (Bruker Daltonik Billerica, Mass. USA).

### Protein identification

To interpret the mass spectra of protein digests for MALDI-TOF analysis, the generated peak lists of the tryptic peptide masses were searched against a non-redundant protein database using MASCOT (Peptide Mass Fingerprint) (Matrix Science Ltd., London, UK, http://www.matrixscience.com/search_form_select.html and Xproteo http://xproteo.com:2698/ for protein identification. Search parameters for MASCOT were: taxonomy, all entities; trypsin cleavage, allow up to one missed cleavage; no restriction on protein mass; peptide mass tolerance 0.2 Da; variable modification, oxidation (M). And searching parameters for Xproteo were: trypsin cleavage, allow up to one missed cleavage, protein mass 0-300 kDa, protein p*I *1-14, peptide mass tolerance 0.2 Da. Proteins were considered valid only if Mowse scores were above the threshold of statistical significance values (95% confidence interval) and d' >= 4 (significant at 99% confidence interval) that were automatically generated by MASCOT and Xproteo are reported in the honeybee genome. The score for the highest ranked hits, or the score of the top matches, to a non-homologous was not reported.

To identify the protein digests for MALDI-TOF-TOF analysis, the generated peaks lists of the tryptic peptide masses were searched against a non-redundant protein database using MASCOT (MS/MS Ion Search) software for protein identification (Matrix Science Ltd., London, UK, http://www.matrixscience.com/search_form_select.html. The searching parameters were set as follows: database, NCBInr; enzyme, trypsin; fixed modifications, carbamidomethyl (C); variable modifications, oxidation (M); no restrictions on protein mass; allow up to one missed cleavage; peptide mass tolerance, ±100 ppm; and fragment mass tolerance, ± 0.2 Da. Proteins were considered valid only Mowse scores were above the threshold of statistical significance values (95% confidence interval) that were automatically generated by MASCOT. The score for the highest ranked hits, or the score of the top matches, to a non-homologous was not reported. The results of the three different search engines were integrated and Mascot was used in the first place, if a protein was identified by one and the other was not included.

The identified proteins were classified according to their biological function by searching GO annotation database http://www.geneontology.org/ and protein knowledge database (UniProt KB) http://www.uniprot.org/.

### Western blot

Following the identification of the protein, to further verify the results of the gel-based comparison mode, we selected major royal jelly protein 1 (MRJP1), MRJP2, MRJP3 showing differentially expressional levels at different phases. Each of MRJP1, MRJP2 and MRJP3 were subjected to 3 replication runs, and 4 μg of protein sample were loaded on each lane separated by stacking (4%) and separating (12%) SDS-PAGE gels. To ensure the specific anti-MRJP1, MRJP2, MRJP3 bands could be detected, protein marker was loaded when running the gels. Gels were run at 120 V for approximately 1.5 h using Mini-Protein II Gel electrophoresis System (Bio-Rad Laboratories Ltd.). Resolved proteins were transferred to a Nitrocellulose transfer membrane (0.2 μm pore size) (Invitrogen) using the iBlot apparatus (Invitrogen, Carlsbad, CA). Nonspecific binding was blocked with 5% (w/v) nonfat milk powder in Tris buffered saline containing 0.1% (v/v) Tween-20 (TBS-T) at room temperature for 1 h. The membranes were then incubated with primary rabbit polyclonal anti-MRJP1, MRJP2, MRJP3 antibodies (developed by our lab) at dilution of 1: 5000 in 2% milk powder in TBS-T at 4°C overnight. Following three washes, the membranes were further incubated with rabbit anti-goat IgG conjugated with horseradish peroxidase (Pierce, Rochford, IL) (1: 10,000 in 2% milk powder in TBS-T), rolling for 1.5 h at room temperature. At the end of this process, the NC membranes were washed for 2 h rolling at room temperature. Immunoreactive protein bands were then visualized by enhanced chemiluminescence detection (ECL, Pierce, Rochford, IL) regents and quantified by densitometry using Quantity-one image analysis system (Bio-Rad Laboratories Ltd.). The human anti-β-actin antibody (1: 5000, sigma) was detected simultaneously as a loading control.

### Biological network analysis

After obtaining the list of the proteins which were differentially expressed on day 1, 3, 6, 12, 15 and 20 from the MALDI-TOF/MS and MALDI-TOF-TOF/MS analysis, a corresponding protein was created from this protein name list. The list was then analyzed by Pathway Studio software (Ariadne Genomics, Rockville, MD). Briefly, the protein list was run against the *Drosophila *database that is equipped with functional relationships from other scientific literature and commercial databases. The filters that were applied included "all shortest paths between selected entities" and "expand pathway". The information received was narrowed down to our protein list of interest in which their involvement and regulatory functions were observed. Protein entities which belong to different functional groups were represented as different shapes according to the default settings of the software, such as "red elipse" for proteins, "sickle" for kinases, "rhomb" for ligands, "stick" for receptors, "O-vertex" for transcription factors, "2 triangles" for phosphates, "sticks" for receptors, "grey elipse" for cell objects, "orange hexagon" and "yellow rectangle" for cell process.

## Abbreviations

HG: hypopharyngeal gland; MRJP: major royal jelly protein; GOD: glucose oxidase; SOD: superoxide dismutase; ADF: actin-depolymerizing factor; PCNA: Proliferating cell nuclear antigen; Rps: ribosome proteins.

## Authors' contributions

FM conducted the experiments and analyzed the data. YF participated in the western blot analysis and database searching for the protein identification. JKL conceived this research, performed biological network analysis and drafted the manuscript. All authors have read and approved the final manuscript.
